# Prognostic value of body mass index before treatment for laryngeal squamous cell carcinoma

**DOI:** 10.7497/j.issn.2095-3941.2015.0043

**Published:** 2015-12

**Authors:** Zhao-Qu Li, Lan Zou, Tian-Run Liu, An-Kui Yang

**Affiliations:** ^1^Sun Yat-sen University Cancer Center, State Key Laboratory of Oncology in South China, Collaborative Innovation Center of Cancer Medicine, Guangzhou 510060, China; ^2^Department of Head and Neck Surgery, Sun Yat-sen University Cancer Center, Guangzhou 510060, China; ^3^Department of Otorhinolaryngology Head and Neck Surgery, The Sixth Affiliated Hospital of Sun Yat-sen University, Guangzhou 510060, China

**Keywords:** Prognosis, nutrition, body mass index (BMI), laryngeal squamous cell carcinoma (LSCC)

## Abstract

**Objective:**

Patients with head and neck cancer often suffer from malnutrition. This study aims to investigate the influence of body mass index (BMI) on the prognosis of laryngeal squamous cell carcinoma (LSCC).

**Methods:**

A total of 473 patients with LSCC initially treated at Sun Yat-sen University Cancer Center between January 2005 and July 2009 were retrospectively reviewed. Survival analysis was performed by the Kaplan-Meier method and Cox regression model.

**Results:**

Low BMI before treatment was significantly associated with poor overall survival in patients with LSCC (*P*<0.001). BMI was an independent prognostic factor for patients with LSCC.

**Conclusion:**

Leanness before treatment was associated with poor prognosis in patients with LSCC. Good nutritional status is favorable to improve survival in patients with LSCC.

## Introduction

Head and neck cancer is the sixth most common cancer worldwide, and the most common malignancy of the head and neck is laryngeal squamous cell carcinoma (LSCC)^1^^,^^2^. This disease often involves male patients older than 50 years old. LSCC seriously affects the life quality of patients, and the 5-year survival is about 60% regardless of stage, treatment strategy, and tumor location^1^^,^^3^.

Several factors are associated with the prognosis of LSCC, including smoking and alcohol consumption, human papillomavirus and Epstein-Barr virus infections, radiation, and heredity^1^^,^^2^. Patients with head and neck cancer often suffer from malnutrition. Nutritional status has been reported to be associated with risk^4^ and survival in several types of head and neck cancer, such as oral^5^^-^^7^ and pharyngeal cancers^8^. Understanding the prognostic value of nutritional status in patients with LSCC bears clinical significance.

Body mass index (BMI), a measure of relative weight based on the mass and height of an individual, is widely used to evaluate nutritional status. BMI has been identified as a possible risk factor for several cancers. Several studies have suggested that high BMI indicates a significantly better prognosis than low BMI for patients with oral cavity and oropharyngeal cancer^5^^,^^6^^,^^8^^,^^9^, whereas other researchers reported conflicting results^7^. However, the influence of BMI on the prognosis of head and neck cancer, including LSCC, remains controversial. In the present study, we investigated the prognostic influence of BMI on LSCC.

## Materials and methods

### Patients and follow up

We retrospectively reviewed 473 patients diagnosed with LSCC and initially treated at Sun Yat-sen University Cancer Center (SYSUCC) between January 2005 and July 2009. The patients consisted of 456 males (96%) and 17 females (4%). Their ages ranged from 24 to 86 years (median, 59 years). The clinical characteristics of the patients are summarized in **Table 1**. No patient had distant metastasis prior to treatment. Among the 473 patients, 430 underwent surgery, 17 received chemotherapy and radiation treatment after surgery, 32 received radiotherapy, and 21 received chemotherapy after surgery. Surgical margins were free of disease.

**Table 1:** Association of BMI status with the clinical characteristics of patients with LSCC

Factors	BMI status (*n*=473)			*P*
	Low, *n* (%)	Medium, *n* (%)	High, *n* (%)	
Age (years)				0.972
≥60	41 (49.4)	144 (48.6)	47 (50.0)	
<60	42 (50.6)	152 (51.4)	47 (50.0)	
Gender				0.320
Male	79 (95.2)	284 (95.9)	93 (98.9)	
Female	4 (4.8)	12 (4.1)	1 (1.1)	
Smoking				0.249
Never	4 (4.8)	11 (3.7)	1 (1.1)	
Former	8 (9.6)	34 (11.5)	17 (18.0)	
Current	71 (85.6)	251 (84.8)	76 (80.9)	
Alcohol				0.056
Never	5 (6.0)	14 (4.7)	1 (1.1)	
Former	48 (57.8)	179 (60.5)	71 (75.5)	
Current	30 (36.2)	103 (34.8)	22 (23.4)	
T stage				0.029
T_1_	25 (30.1)	125 (42.2)	51 (54.2)	
T_2_	19 (22.9)	75 (25.3)	20 (21.3)	
T_3_	21 (25.3)	49 (16.6)	14 (14.9)	
T_4a_+T_4b_	18 (21.7)	47 (15.9)	9 (9.6)	
N stage				<0.001
N_0_	47 (56.6)	234 (79.1)	82 (87.2)	
N_1_	15 (18.1)	27 (9.1)	3 (3.2)	
N_2a_+N_2b_+N_2c_	17 (20.5)	32 (10.8)	9 (9.6)	
N_3_	4 (4.8)	3 (1.0)	0 (0)	
Pathology grade				0.177
Well	41 (49.4)	159 (53.7)	62 (66.0)	
Moderate	33 (39.8)	111 (37.5)	24 (25.5)	
Poor	9 (10.8)	26 (8.8)	8 (8.5)	
Tumor location				0.042
Supraglottis	29 (34.9)	74 (25.0)	15 (15.9)	
Glottis	53 (63.9)	214 (72.3)	78 (83.0)	
Subglottis	1 (1.2)	8 (2.7)	1 (1.1)	
Total	83	296	94	

BMI, body mass index; LSCC, laryngeal squamous cell carcinoma.

Of the 43 patients who did not undergo surgery, 11 received radiation treatment, 22 underwent chemotherapy, and 10 underwent both chemotherapy and radiation treatment.

The survival time was defined as the period from the date of the initial treatment to the date of death or final follow up (July 2014). This study followed the Declaration of Helsinki on medical protocol and ethics, and it was approved by the Institutional Review Board at SYSUCC. Written informed consent was obtained from all patients for the publication of this study.

### Criteria for nutritional status

The nutritional status of the patients was classified by BMI in accordance with Asian-specific BMI cutoff points as follows: overweight (≥25 kg/m^2^), normal weight (≥18.5 and <25 kg/m^2^), or underweight (<18.5 kg/m^2^)^10^. The BMI data were obtained before treatment.

### Statistical analysis

The Chi-square test and Kruskal-Wallis H test were used for bivariate analysis. Kaplan-Meier and log-rank tests were used for survival analysis. Multivariate Cox regression analysis was performed for significant variables found using univariate analysis. SPSS 16.0 was used for all analyses. *P*<0.05 was considered statistically significant.

## Results

### Patient characteristics and bivariate analysis

A total of 473 patients participated in this study. The clinicopathological characteristics of the patients are summarized in **Table 1**. Among the 473 patients, 83 (17%) were underweight, 296 (63%) were of normal weight, and 94 (20%) were overweight based on their BMI. The groups of patients with different BMI statuses showed similar distributions of host and tumor factors, including age, gender, smoking and alcohol intake, and pathology grade. Patients with low BMI showed a high incidence of advanced T stage (47.0% *vs.* 32.5% *vs.* 24.5%, *P*=0.029) and N stage (25.3% *vs.* 11.8% *vs.* 9.6%, *P*<0.001; **Table 1**).

All patients were divided into the survival and non-survival groups. The survival group consisted of 332 patients (70%), and the non-survival group comprised 141 patients (30%). The bivariate analysis of factors between the groups is presented in **Table 2**. The two groups showed significant differences in age, smoking and alcohol intake, T stage, N stage, pathology grade, treatment strategy, tumor location, and BMI status. However, no significant difference was found in gender between the two groups.

**Table 2:** Bivariate descriptive analysis of patients with LSCC

Factors	No. of patients (%)			*P*
	Total (%)	Survival group	Non-survival group	
Age (years)				0.003
≥60	232 (49)	148 (45)	84 (60)	
<60	241 (51)	184 (55)	57 (40)	
Gender				0.264
Male	456 (96)	318 (96)	138 (98)	
Female	17 (4)	14 (4)	3 (2)	
Smoking				0.494
Never	16 (3)	13 (4)	3 (2)	
Former	59 (13)	39 (12)	20 (14)	
Current	398 (84)	280 (84)	118 (84)	
Alcohol				0.034
Never	20 (4)	16 (5)	4 (3)	
Former	298 (63)	219 (66)	79 (56)	
Current	155 (33)	97 (29)	58 (41)	
T stage				<0.001
T_1_	201 (42)	176 (53)	25 (18)	
T_2_	114 (24)	77 (23)	37 (26)	
T_3_	84 (18)	45 (14)	39 (28)	
T_4a_+T_4b_	74 (16)	34 (10)	40 (28)	
N stage				<0.001
N_0_	363 (77)	292 (88)	71 (50)	
N_1_	45 (10)	18 (5)	27 (19)	
N_2a_+N_2b_+N_2c_	58 (12)	22 (7)	36 (26)	
N_3_	7 (1)	0 (0)	7 (5)	
Pathology grade				<0.001
Well	262 (55)	209 (63)	53 (38)	
Moderate	168 (36)	107 (32)	61 (43)	
Poor	43 (9)	16 (5)	27 (19)	
Treatment				<0.001
Surgery alone	363 (77)	284 (86)	79 (56)	
Surgery + radiotherapy	33 (7)	20 (6)	13 (9)	
Surgery + chemotherapy	16 (3)	6 (1)	10 (7)	
Surgery + chemoradiotherapy	18 (4)	10 (3)	8 (6)	
Chemoradiotherapy	24 (5)	12 (4)	12 (9)	
Chemotherapy	19 (4)	0 (0)	19 (13)	
Tumor location				0.001
Supraglottis	118 (25)	67 (20)	51 (36)	
Glottis	345 (73)	259 (78)	86 (61)	
Subglottis	10 (22)	6 (2)	4 (3)	
BMI				<0.001
High	94 (20)	82 (25)	12 (9)	
Medium	296 (63)	224 (67)	72 (51)	
Low	83 (17)	26 (8)	57 (40)	
Total	473	332	141	

LSCC, laryngeal squamous cell carcinoma; BMI, body mass index.

### Prognostic value of BMI

**Figure 1:**
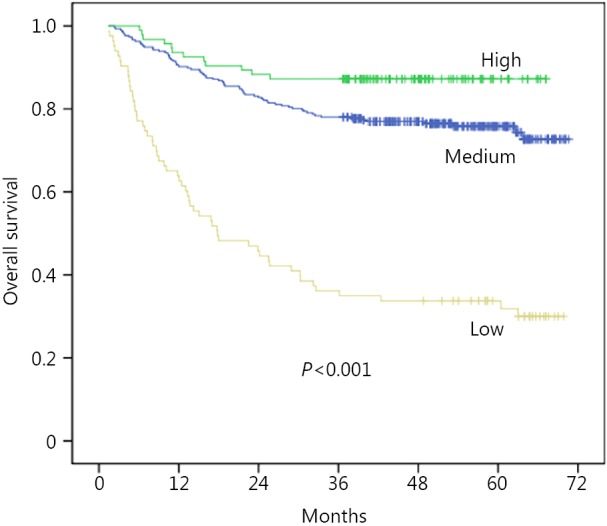
Kaplan-Meier curves for overall survival according to BMI.

The overall 5-year survival rate of these patients was 72.3%. **Figure 1** reveals that the underweight patients had the lowest survival, whereas the overweight patients had the best survival (*P*<0.001). The 5-year overall survival rates of the overweight group, normal weight group, and underweight group were 87.2%, 78.0%, and 34.9%, respectively. The median survival time of the underweight group was 29.6 months.

The Cox proportional hazards model was used to verify whether the BMI status and other variables are independent prognostic factors for LSCC patients. Univariate analysis showed that age, history of smoking and alcohol intake, T stage, N stage, pathology grade, tumor location, treatment strategy, and BMI were associated with survival in patients with LSCC. Multivariate analysis of these variables showed that age, history of smoking and alcohol intake, T stage, N stage, pathology grade, treatment strategy, and BMI were independent prognostic factors for patients with LSCC, and low BMI was significantly associated with poor prognosis (**Table 3**).

**Table 3:** Univariate and multivariate Cox regression analysis for overall survival in patients with LSCC

Factors	Univariate analysis			Multivariate analysis	
	HR (95% CI)	*P*		HR (95% CI)	*P*
Age	1.658 (1.184-2.321)	0.003		1.854 (1.297-2.650)	0.001
Gender	0.540 (0.172-1.695)	0.291			
Smoking	0.821 (0.547-1.231)	0.340			
Alcohol	1.247 (0.953-1.633)	0.108			
T stage					
T_1_	Ref			Ref	
T_2_	2.862 (1.723-4.574)	<0.001		1.852 (1.060-3.233)	0.030
T_3_	4.881 (2.953-8.069)	<0.001		2.535 (1.442-4.454)	0.001
T_4a_+T_4b_	6.207 (3.761-10.244)	<0.001		2.842 (1.585-5.096)	<0.001
N stage					
N_0_	Ref			Ref	
N_1_	4.183 (2.683-6.523)	<0.001		2.077 (1.269-3.401)	0.004
N_2a_+N_2b_+N_2c_	4.681 (3.130-7.003)	<0.001		2.088 (1.263-3.452)	0.004
N_3_	25.230 (11.173-56.972)	<0.001		7.659 (2.919-20.102)	<0.001
Pathology grade					
Well	Ref			Ref	
Moderate	2.060 (1.430-2.969)	<0.001		1.497 (1.010-2.219)	0.044
Poor	4.133 (2.564-6.661)	<0.001		1.791 (1.047-3.064)	0.033
Tumor location	0.562 (0.401-0.787)	0.001		1.197 (0.847-1.691)	0.308
Treatment	1.540 (1.407-1.687)	<0.001		1.259 (1.123-1.413)	<0.001
BMI	1.964 (1.616-2.388)	<0.001		1.747 (1.433-2.130)	<0.001

LSCC, laryngeal squamous cell carcinoma; BMI, body mass index.

## Discussion

Our study demonstrated that BMI was significantly associated with the prognosis of LSCC, and low BMI before treatment predicted poor prognosis in patients with LSCC.

Malnutruitional status is reportedly associated with poor prognosis in several types of cancers^4^^-^^8^^,^^11^. In these studies, leanness was found to be associated with increased risk for head and neck cancer regardless of the smoking and drinking status, and it was reported as an early marker of oral and pharyngeal cancer^4^^-^^8^. For example, oral cancer patients with a BMI <22.8 kg/m^2^ have poor prognosis, and weight loss can predict poor prognosis in recurrent oral and oropharyngeal carcinoma^5^^,^^6^. A previous retrospective analysis reported that patients with head and neck cancer and preoperative weight loss greater than 5% show poor outcome^11^. Notably, a study by Iyengar *et al.*^7^ indicated that obesity is an independent predictor of increased risk of death for patients with early-stage oral tongue cancer. These conflicting results may be due to an inadequate sample size and inconsistent BMI cutoff values. These studies also found various tumor sites in the oral cavity, oropharynx, and larynx with various types of histopathology. In the current study, a total of 473 patients diagnosed with LSCC were analyzed, and the patients were classified according to Asian-specific BMI cutoff values. We found that low BMI was significantly associated with poor survival in LSCC, which was in agreement with previous data.

Malnutrition commonly occurs in patients with head and neck cancer, particularly in laryngeal cancer, which seriously affects the quality of life and nutritional status of the patient. Thus, weight loss before treatment is associated with poor prognosis. In China, underweight patients are associated with low income and education. These patients are less likely to receive proper treatment at an early stage. In addition, good nutritional status can improve survival by strengthening immunity and helping patients develop a high tolerance for extended therapeutic periods. However, our conclusion requires further demonstration. We found that patients with low BMI showed an increased incidence of advanced T and N stages, which may also contribute to decreased survival in patients with low BMI.

The present study demonstrated that patients over 60 years old exhibited low survival rates. Aging patients with oral tongue cancer have been reported to have high disease-speciﬁc mortality^12^. This finding suggested increased vulnerability to deterioration of nutritional or physical status among old patients. Heavy tobacco and alcohol consumption were determined to be adverse prognostic factors in patients with head and neck cancer, including laryngeal cancer^13^. Another study identified heavy alcohol consumption as a risk factor for malnutrition^14^. Postoperative complications have also been reported to be associated with poor prognosis in patients with oral cancer^15^. The present study found similar factors that could contribute to the advanced disease stage or poor general condition, both of which could increase the risk of developing complications. Developing complications would inevitably delay the administration of standard therapy, leading to recurrence or distal metastasis. Recurrence or distal metastasis may negatively affect initial treatment. Consequently, patients with low BMI have poor prognosis^5^.

The molecular and cellular mechanisms by which BMI affects the prognosis of cancer are complicated and unclear. Several studies have shown that a speciﬁc enteral nutrition formula containing n-3 fatty acid can prevent the deterioration of nutritional status during chemoradiation therapy for head and neck cancer; triglycerides have also been reported to promote cancer cell proliferation, and this anti-apoptotic activity is probably due to the generation of reactive oxygen species and oxidative stress, which leads to DNA damage^16^^,^^17^. Levels of adipokine, leptin, and adiponectin influence obesity and several signal transduction pathways involved in cell survival^18^. Another study reported that obesity induces insulin-resistance through the accumulation of macrophages that secrete inflammatory mediators, such as tumor necrosis factor-α, interleukin-6, and prostaglandin E2^19^. The insulin receptor activating signaling pathways are also associated with invasion and metastasis of malignant tumors^20^. However, the mechanisms require further study.

## Conclusion

Our study proved that BMI was an independent prognostic factor for patients with LSCC, and low BMI before treatment could predict poor prognosis for patients with LSCC. Good nutritional status could improve survival in patients with LSCC. However, our study was limited by the retrospective design. Thus, further studies are necessary to verify the correlation between BMI and the prognosis of LSCC.
